# Images: Hajdu–Cheney syndrome

**DOI:** 10.4103/0971-3026.40298

**Published:** 2008-05

**Authors:** H Satishchandra, SD Madhu, KR Prasad

**Affiliations:** Department of Radio Diagnosis, Bowring and Lady Curzon Hospitals, Bangalore Medical College and Research Institute, Bangalore, India

Hajdu and Kauntze described a rare disorder called Hajdu–Cheney syndrome (HCS) for the first time in 1948.[1] Over 50 cases have been reported till date.[2] Some were reported under the terms acro-osteolysis[3] and arthrodento-osteodysplasia.[4] HCS is a unique bone metabolism disorder, characterized clinically by short stubby fingers, a peculiarly shaped skull, premature loss of teeth, and short stature.[5] The clinical presentation, biochemical investigations, and typical radiological features aid in confirming the diagnosis.

## Case Report

An 18-year-old lady was referred for an orthopantomogram (OPG). She was then reviewed by the dental, pediatric, and radiology departments and after appropriate investigations was diagnosed to have HCS.

**Figure 1:**
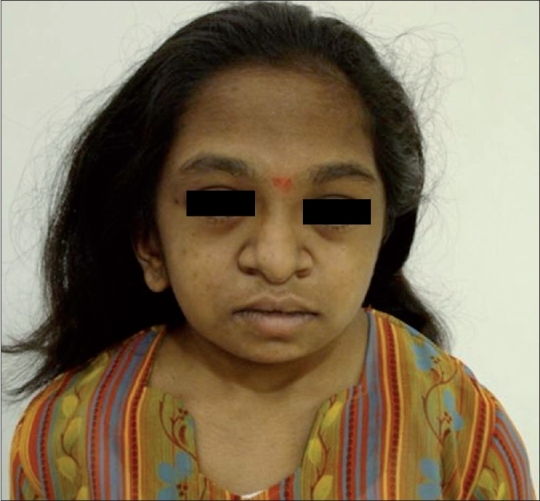
A frontal photograph shows the characteristic facies

Clinical examination revealed short stature, coarse features, bushy eyebrows, anti-Mongoloid slant [Figure 1], flared anteverted nostrils, dental abnormalities, short stubby fingers, a wide short neck, genu valgus, and swollen feet with short toes. All her other systemic examinations were normal. Blood tests, urinalysis, and serum chemistries, along with screening tests for metabolic and genetic disorders were normal. A review of the patient's family history showed that her parents and four siblings were apparently normal.

**Figure 2:**
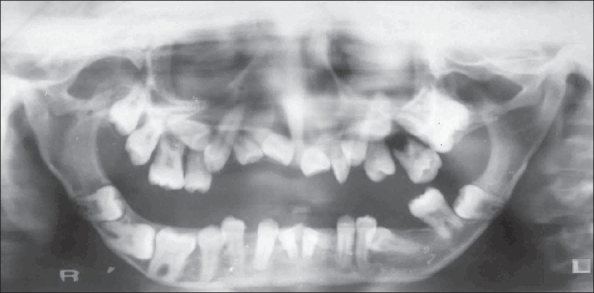
An OPG shows hypoplastic maxillary and mandibular bones, widened mandibular angles, and unerupted and maloccluded tooth

**Figure 3:**
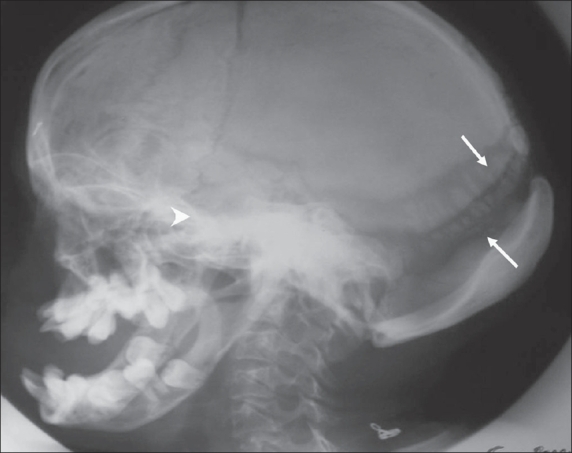
A lateral skull radiograph shows Wormian bones (arrows), widened sutures, an enlarged pituitary fossa (arrowhead), basilar invagination, and a hypoplastic frontal sinus

**Figure 4:**
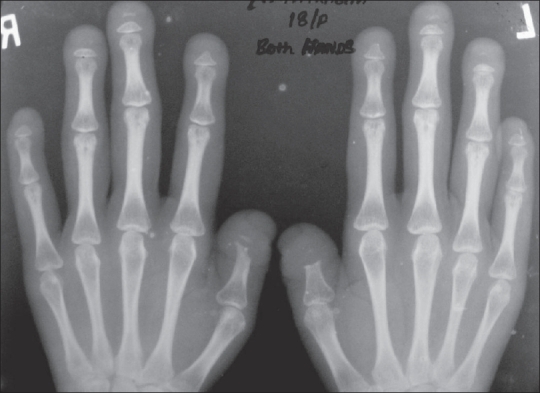
A frontal radiograph of both hands shows acro-osteolysis

**Figure 5:**
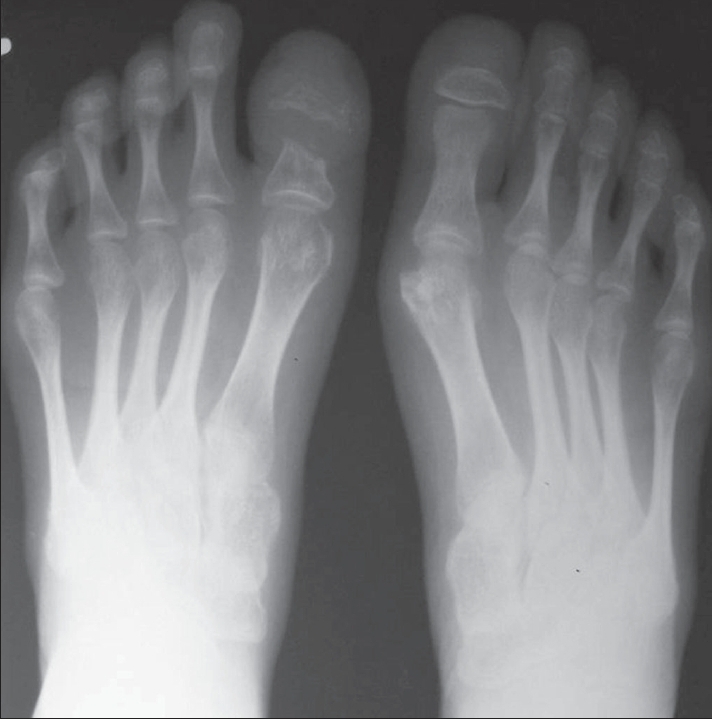
A frontal foot radiograph shows acro-osteolysis of the distal phalanges of both great toes, with resorption of the proximal phalanx of the right great toe

**Figure 6:**
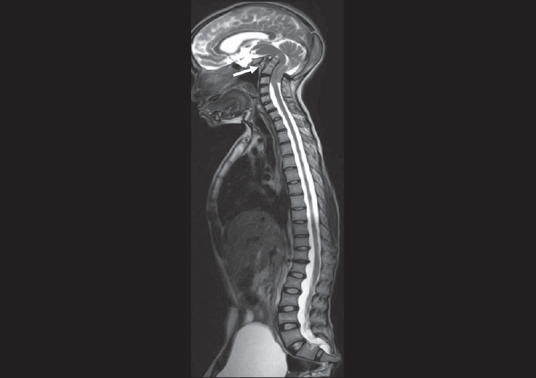
A T2W sagittal whole spine image shows basilar invagination (arrow), with a widened spinal canal throughout the dorsal and lumbar spine

**Figure 7:**
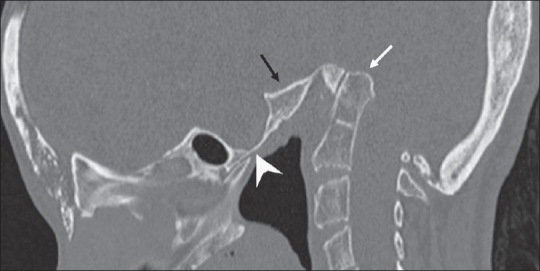
A sagittal CT scan sagittal images shows basilar invagination (arrow), enlarged pituitary fossa (arrowhead), and a deformed clivus (black arrow)

**Figure 8:**
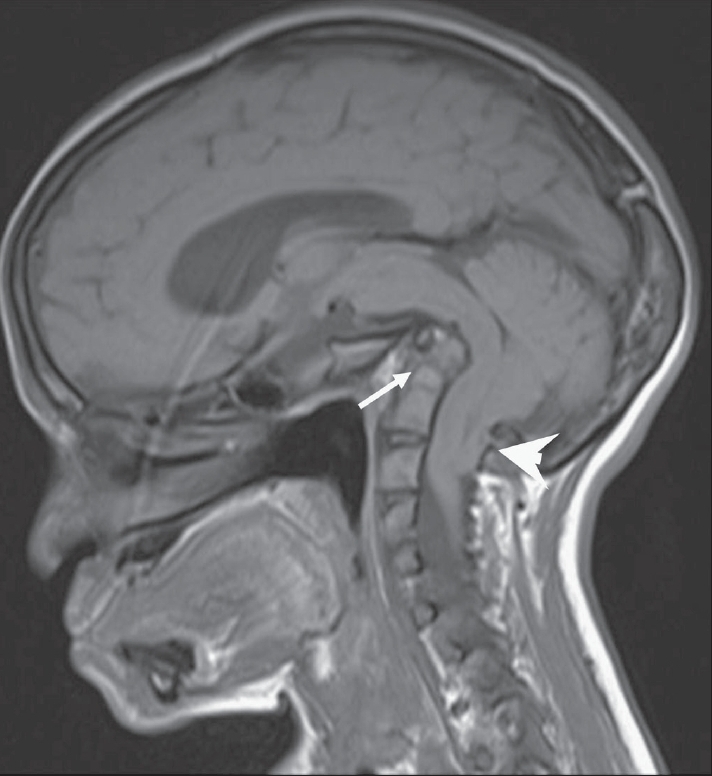
Sagittal T1W MRI shows basilar invagination (arrow) with an Arnold-Chiari I malformation (arrowhead)

The OPG [Figure 2] showed hypoplasia of the maxilla, mandible, and mandibular condyles, a widened mandibular angle, and unerupted and maloccluded teeth. The skull radiograph [Figure 3] revealed Wormian bones; widened sutures, predominantly involving the lambdoid; an enlarged pituitary fossa; basilar invagination; a steep clivus; and hypoplastic frontal sinuses. There was transverse osteolysis of the terminal phalanges with overlying soft tissue swelling [Figure 4], along with osteolysis of the great toes on both sides, with resorption of the proximal phalanx of the 1^st^ right toe [Figure 5]. There were normal clavicles, a ‘heart-shaped’ pelvic inlet, and bilateral genu valgum, along with dorso-lumbar kyphoscoliosis and widening of the spinal canal, with posterior scalloping in the dorsal and lumbar regions [Figure 6]. Basilar invagination with an Arnold–Chiari I malformation [Figures 7 and 8], a deformed clivus with hypoplastic mandibular condyles, and an enlarged pituitary fossa were also seen.

## Discussion

HCS is a rare bone disease with a defect in collagen and bone formation.[3,6] It has an autosomal dominant inheritance[6] but sporadic cases have also been reported.[5] It presents at birth with dysmorphic features. With advancing age, the features become more obvious. The distinctive facies with frontal bossing, mid-facial flattening, coarse thick hair, and low-set ears with large ear lobules[1,6,7] should alert the physician. Once suspected, the characteristic hand findings make the diagnosis easy. HCS has distinctive features with specific and supportive signs.

The specific findings are:

1. Acro-osteolysis of the distal phalanges of the hands and feet
2. Widened cranial sutures, particularly the lambdoid
3. Wormian bones

The presence of acro-osteolysis with any three other features, such as Wormian bones, open skull sutures, platybasia, micrognathia, mid-facial flattening, premature loss of teeth, coarse hair, and short stature, is enough for a diagnosis.[4] In adults, acro-osteolysis and a positive family history of HCS are sufficient for a positive diagnosis of this condition. All these findings were seen in our patient.

The supportive, but less distinctive, features include hypoplastic alveolar arches with loss of teeth and unerupted teeth, hypoplastic mandible and maxilla, a widened sella turcica, basilar invagination with its complications, kyphoscoliosis, short stature, and genu valgum.[2,6–8] All these findings were also present in our case.

The acro-osteolysis has been variably attributed to either a vascular etiology[6] or defective development[4] leading to pseudo-osteolysis.

The differential diagnoses include cleidocranial dysplasia (associated with abnormal/absent clavicle), pyknodysostosis (an osteosclerotic condition), progeria (a syndrome of premature aging), and Rothmund–Thompson syndrome (a hereditary, benign skin condition with a multitude of findings quite distinct from those seen in HCS).

The treatment of the disorder is symptomatic and the prognosis is quite good, with the morbidity depending on the bone changes caused by acro-osteolysis and the neurological complications caused by basilar invagination.
